# Evaluation and Management of Unexpected Functional Rudimentary Uteri in Mayer–Rokitansky–Küster–Hauser Syndrome of Chinese Women

**DOI:** 10.1155/2020/6808409

**Published:** 2020-11-24

**Authors:** Shan Deng, Lan Zhu, Qinjie Tian

**Affiliations:** Department of Obstetrics and Gynecology, Peking Union Medical College Hospital, Chinese Academy of Medical Sciences, National Clinical Research Center for Obstetrical and Gynecological Diseases, Beijing, China

## Abstract

**Objective:**

To elucidate the characteristics of symptomatic attack of rudimentary uteri in MRKH syndrome and highlight the rare and unexpected possibilities.

**Methods:**

A cohort of 202 Chinese MRKH syndrome patients admitted to the Peking Union Medical College Hospital from Jan 2009 to Dec 2016 was analyzed retrospectively. Based on the symptoms of abdominal pain before vaginoplasty, the patients were categorized into the asymptomatic and symptomatic groups.

**Results:**

Totally, 21 patients had their uteri removed due to obstructive bleeding, 19 of them had symptoms of abdominal pain before vaginoplasty, the mean duration of abdominal pain before artificial vaginoplasty was 5.0 years (range, 0.5–10 years), and the mean age at first onset of recurrent abdominal pain was 14.3 years old (range 11–18). Two special cases showed unusual long incubation periods up to 23 years. Ultrasound detected endometrioid echo in four asymptomatic patients. Among the symptomatic group, 7 patients had no imaging evidence for endometrial cavities despite clinical pain. Two of them developed severe symptoms over the next two or four years and eventually had their uteri removed. Two patients reported persistent abdominal pain with a visual analog scale (VAS) score of 4–5, still under observation. Three patients were lost to follow-up.

**Conclusion:**

More than 10% of the patients with MRKH syndrome had surgical indication to remove the rudimentary uteri. The discrepancy between clinical symptoms and imaging calls for the vigilance for prophylactic surgery or prolonged follow-up.

## 1. Introduction

The Mayer–Rokitansky–Küster–Hauser (MRKH) syndrome, also known as Müllerian aplasia, refers to the congenital absence of the upper two-thirds of the vagina with a rudimentary uterus or even without a uterus [1], which affects at least 1 in 4000 to 5000 female live births [2]. The syndrome is characterized by primary amenorrhea with normal secondary sexual characteristics, normal ovarian functions, and a normal karyotype of 46,XX [3–5]. The typical MRKH syndrome presents as isolated agenesis or hypoplasia of Müllerian ducts with normal ovaries and kidneys, whereas atypical MRKH syndrome manifests as ovarian and/or renal malformation together with anomalies of the Müllerian structures. Vaginoplasty is the main therapy to resolve problems regarding sexual intercourse; however, the patients are unable to conceive except through surrogacy or uterine transplantation.

It is well known that patients with MRKH syndrome have no uterus or only the primordial uterus. However, despite the functional silence, approximately 40% of the patients with MRKH syndrome have a rudimentary uterus with endometrium [6]. Approximately 7%–10% of these patients could have similar clinical symptoms of menstrual periods without bleeding [7]. Some reports even suggested that some rudimentary uteri of patients with MRKH syndrome may potentially restore unimpeded menstrual flow by surgical reestablishment of uterovaginal continuity [8, 9]. Two special cases of long latent (with an interval of 23 and 8 years between previous vaginoplasty and hysterectomy) functional rudimentary uteri of MRKH syndrome were reported in this paper, and a retrospective cohort study of 202 cases of MRKH was designed to elucidate the characteristics of the symptomatic rudimentary uterus in MRKH syndrome and to highlight the rare and unexpected possibilities of delayed attack, which is worthy of attention.

## 2. Materials and Methods

### 2.1. Patient Evaluation

We reviewed and analyzed 202 cases of patients with MRKH syndrome admitted to the Department of Obstetrics and Gynecology, Peking Union Medical College Hospital (PUMCH), Chinese Academy of Medical Sciences, from January 1, 2009, to December 31, 2016. All patients met the diagnostic criteria of MRKH syndrome, including primary amenorrhea, no vagina, normal female secondary sexual characteristics, normal ovarian functions, and 46,XX karyotype. Exclusion criteria included abnormal karyotypes, secondary amenorrhea, and vaginal atresia with hematometra and hematocolpos.

The study protocol was approved by the local ethics committee at the authors' affiliated institution, and patient consents were obtained preoperatively.

Demographic and baseline data, including patient's age, personal and family history, steroid hormone levels, and karyotype, were obtained. All patients underwent gynecologic and routine preoperative examinations, including pelvic ultrasound. Transabdominal or transrectal two-dimensional ultrasonography was performed using color Doppler ultrasound diagnosis systems and a 5-9 MHz transrectal probe (iU22, Philips, Best, The Netherlands; Logiq 9, GE Healthcare, Waukesha, WI, USA) by experienced ultrasound doctors. Seventeen patients also underwent pelvic magnetic resonance imaging (MRI) using 3 T scanners (Sigma, GE Healthcare, Waukesha, WI, USA; Ingenia 3.0T CX; Philips, Best, the Netherlands).

Based on the presence of periodic or irregular abdominal pain before vaginoplasty, the patients were categorized into the symptomatic and asymptomatic groups in order to compare the proportion of functional rudimentary uteri and surgical management.

### 2.2. Statistical Analysis

Data were expressed as numbers and percentages. Statistical analysis was performed with IBM SPSS Statistics 19.0 (IBM Corporation, Somers, NY, USA). All statistical tests in this study were two-sided. Estimates were evaluated at the 5% significant level, and 95% confidence intervals (CIs) were provided.

## 3. Results

### 3.1. Demographic Characteristics and Surgical Methods

**Figure 1:**
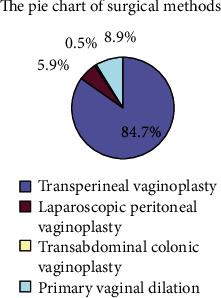
The pie chart of surgical methods.

**Figure 2:**
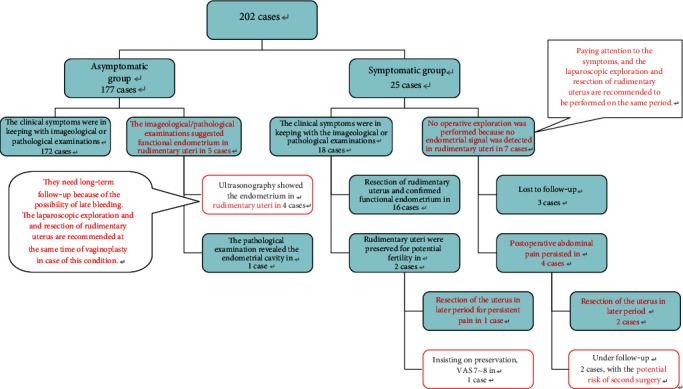
The clinical events' flowchart.

Two hundred and two patients were eligible for this retrospective analysis. The mean age of patients at vaginoplasty was 24.3 years (range, 12–42 years). Based on our classified criteria, there were 177 (87.6%) and 25 (12.4%) patients in the asymptomatic and symptomatic groups, respectively. There was no significant difference in age between the two groups. The proportion ratio of surgical methods for vaginoplasty was showed in a pie chart (Figure 1). In the asymptomatic group, most patients did not undergo pelvic exploration except those with peritoneal approach for vaginoplasty (12 cases, 5.9%). In contrast, the majority of the symptomatic group required hysterectomy to relieve the obstructive pain except 7 cases without definite endometrial signal in rudimentary uteri.  Figure 2 shows the courses of clinical events.

### 3.2. Rudimentary Uteri with Clinical Symptoms

Totally, 21 patients had their uteri removed due to obstructive bleeding, only one symptomatic patient insisted on preserving her rudimentary uterus and hoping for assisted reproduction. Two special cases therein from the asymptomatic group had unusual long preclinical periods with 23 and 8 years, respectively, after vaginoplasty and got symptom relieved by hysterectomy eventually. The other 19 patients who had hysterectomy all belonged to the symptomatic group; apart from two patients who had delayed hysterectomy because of atypical symptom as well as no imaging evidence of endometrial cavity before vaginoplasty, 17 patients had their rudimentary uteri removed at the time of vaginoplasty. The endometrial cavities were confirmed by pathological examination in all patients except one regardless of its enlarged appearance.

There were 5 patients in the symptomatic group who also had atypical or mild abdominal pain but did not remove the uteri because no ultrasonic sign of endometrial cavity was detected by the time of vaginoplasty. Three of them were lost to follow-up, and the other two patients were still under observation.

The mean duration of abdominal pain before vaginoplasty was 5.0 years (range, 0.5–10 years). The mean age at first onset of recurrent abdominal pain was 14.3 years old (range 11–18).

### 3.3. Discrepancy between Clinical Symptoms and Images

Among the asymptomatic patients, endometrioid echo had been detected by ultrasound in four patients, but symptomatic silence continued nowadays. One rudimentary uterus was detected endometrial cavity by pathological examination occasionally.

Among the symptomatic group, 7 patients had no imaging evidence for endometrial cavities despite clinical pain. Two of them developed severe symptoms after vaginoplasty two or four years later and eventually had their uteri removed. Two patients reported persistent abdominal pain with a visual analog scale (VAS) score of 4–5, but still under observation. Three patients were lost to follow-up.

### 3.4. Two Special Cases of Delayed Symptomatic Attack

**Figure 3:**
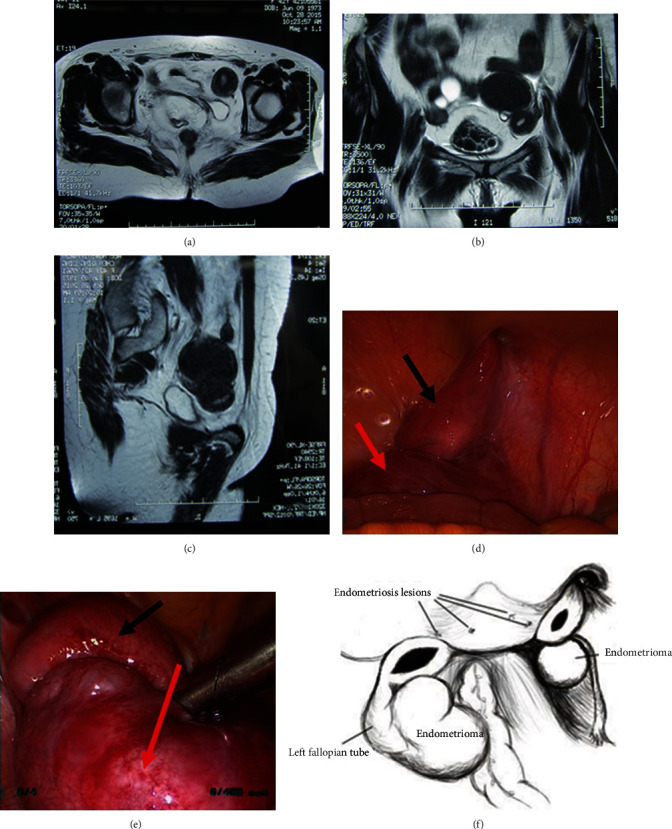
(a–c) MRI shows bilateral rudimentary uteri with endometrium and bilateral low-signal cystic mass of ovaries; (d) right rudimentary uterus adjacent to the internal inguinal ring; (e) left rudimentary uterus and left ovarian chocolate cyst covered by mesosalpinx; (f) diagrammatic drawing of the operational finding: bilateral residual uteri (maximum diameter, 6–7 cm on the left and 2–3 cm on the right), bilateral ovarian chocolate cysts. Black arrows indicate the uteri, and red arrows point to the left ovarian cyst.

Case 1: a 42-year-old woman underwent transabdominal colonic vaginoplasty at the age of 19 years, and surgical records did not describe the enlarged uterus. She had periodic abdominal pain since the age of 30 years. A pelvic mass was noted because of more than 1-year history of abdominal distention. Preoperative ultrasonography showed that the uterine structure was not detected behind the bladder. The left ovary was unclear, and anechoic lesion with a size of 5.5 × 5.2 cm was found in the left adnexal region, which was full of punctate hypoecho, and no clear blood flow was observed by color Doppler flow imaging (CDFI). A hypoechoic nodule could be seen outside the mentioned anechoic lesion, which was approximately 2.9 × 2.3 × 1.8 cm in size, and a 0.3 cm thick endometrial echo was found. The right ovary was 5.2 × 2.7 cm, with a 3.0 × 2.2 cm anechoic cyst. Another 1.6 × 1.0 cm hypoechoic nodule could be seen in the left inguinal region, nonhomogeneous and strong punctate echoes were present, and a minimal blood flow signal could be found by CDFI. Pelvic magnetic resonance imaging (MRI) was also performed (Figure 3). Laparoscopic exploration revealed bilateral functional rudimentary uteri and ectopic cysts in the ipsilateral ovary, and pain was relieved completely after removal of hematometra, hematosalpinx, and ovarian endometrial cysts.

**Figure 4:**
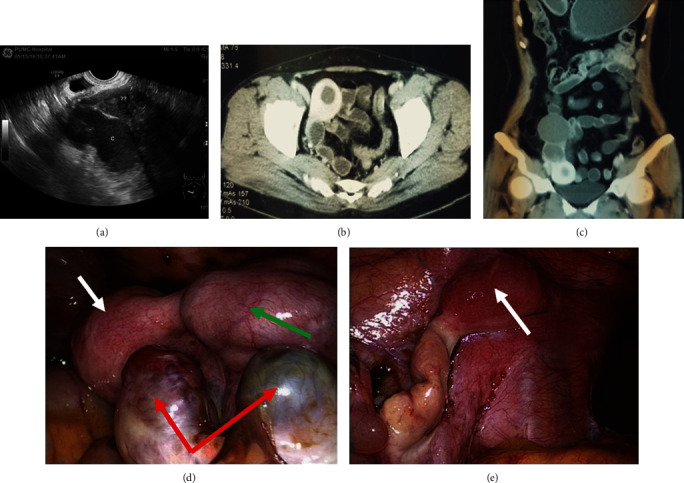
(a) Preoperative ultrasonography: a hypoechoic lesion of 4.2 × 3.9 × 3.1 cm was found in the right adnexal region, in which there was an isoecho lesion of 1.5 × 0.7 cm. Circumferential blood flow signal was observed by CDFI. An anechoic cyst of 7.2 × 3.9 × 3.8 cm was also seen outside the hypoechoic lesion mentioned above. (b, c) MRI shows bilateral residual uterus with the endometrium in the right, and a segmental low-signal cystic lesion is located on the upper right of the right uterus. The solid nodule on the left was in the inner wall of the ileum. (d, e) Laparoscopic findings. Bilateral residual uteri of maximum diameter (d) 5-6 cm on the right and (e) 2–3 cm on the left, fallopian tubes, and right ovarian chocolate cysts. The white arrow points to the uteri. The red arrow points to the ovarian cyst. The green arrow points to the swollen fallopian tube with hematocele.

Case 2: a 30-year-old woman underwent transperineal vaginoplasty with amniotic membrane. She was satisfied with her sex life after surgery; however, periodic abdominal pain appeared and gradually worsened after several months of operation (Figure 4). Bilateral residual uteri and right ovarian endometriosis cyst were removed 8 years after vaginoplasty. Pathological examination confirmed proliferative changes of the endometrium in the right rudimentary uterus, and no endometrium was detected in the left muscular nodule by visual observation and pathological diagnosis.

## 4. Discussion

Our study showed that at least 10.4% (21/202 in our series) of patients with MRKH syndrome had surgical indication to remove the rudimentary uteri. Although the vast majority of patients show signs of functional endometrium before vaginoplasty, some patients would experience unusually long latent period. 5.9% (5 + 7/202) of the patients, who showed discrepancy between clinical symptoms and ultrasound even MRI image, should call for vigilance for follow-up.

Based on the practical experience at our hospital, 90.4% of the patients were properly treated by transperineal artificial vaginoplasty with biological patch [10]. However, despite the functional silence, approximately 40% of the patients with MRKH syndrome have a rudimentary uterus with endometrium [6]. In order to identify the functionality of rudimentary uterus, the painful symptoms related to blocked bleeding seemed to be an important clue. Approximately 7%–10% of the MRKH patients could have clinical symptoms^7^. The cumulative incidence of functional rudimentary uteri in our cohort was at least 10.4% (21/202), regardless of three patients that were lost to follow-up, two patients with ambiguous symptoms who were still under observation, and one patient with unusual persistence, which was slightly higher than the number reported in previous literature [7, 11]. The presence of the functional endometrium was consistently verified in all symptomatic patients who underwent removal or anastomosis of rudimentary uteri. In view of the long latent period of two special cases, the rate of symptomatic attack of the rudimentary uteri has the possibility to increase over time with patients' ages or prolonged follow-up.

On the other hand, based on the literatures published by radiologists, MRI could detect the endometrium and/or hematometra in 9.8% of the patients [12], which means that MRI may also miss some functional rudimentary uteri. Although MRI assessment is more sensitive and accurate than ultrasonography [6, 13], there was no difference between ultrasound and MRI results in our series of 17 cases (not rendered in Results). The symptoms and signs of blocked uterine bleeding seemed to be more important and sensitive to indicate the presence of a functional endometrium. It is essential to combine the medical history, clinical manifestations, pathological examinations, and long-term follow-up to have a proper treatment.

Cases 1 and 2 were initially judged as asymptomatic in the early stage, but finally diagnosed as functional rudimentary type after many years. Based on our retrospective analysis, the vast majority of patients who had functional rudimentary uteri would present pain of blocked bleeding before they underwent vaginoplasty. Speculated by the onset of abdominal pain, the estimated age of menarche in most cases (14.3 years old, range 11-18) seemed to be similar with the normal population or just a few years late. In other words, the latencies of the functional endometrium in most rudimentary uteri were not long. However, two special cases, as well as similarly reported cases [14], reminded us that the hormone response of rudimentary uteri might change over time and finally result in blocked bleeding symptoms [15]. Such similar changes also appeared in our studies of other types of uterine malformation, such as unicornuate uterus, in which a noncommunicating rudimentary uterus could also present with bleeding 15 years after menarche (not reported).

**Figure 5:**
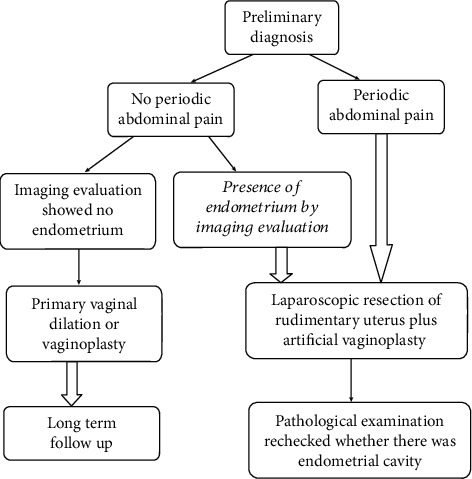
Diagnostic and therapeutic flow charts of MRKH syndrome in PUMCH.

The most intriguing issue is that 5.9% of the patients (5 out of 176 asymptomatic patients and 7 out 26 symptomatic patients) showed discrepancy between clinical symptoms and imaging. The symptomatic patients should be carefully examined during consultation and recommended prophylactic laparoscopic hysterectomy. The asymptomatic patients without resection of the rudimentary uteri, particularly those with endometrial signal detected by ultrasound, should have a long-term follow-up and be informed of the possibility of a second time surgery when making the informed choice of surgical methods. The recommended diagnosis and management procedure at our center are illustrated in Figure 5. The combination of the image examination and clinical symptoms was essential to estimate the functional status of the rudimentary uteri. If periodic abdominal pain was present regardless of whether the endometrium is shown by imaging examination, laparoscopic exploration and resection of the rudimentary uteri were recommended to avoid the risk of repeated surgeries. If there was no pain and no positive finding was found in the image examination, it was appropriate to perform simply vaginoplasty alone, but long-term follow-up is also recommended.

In summary, the functional potential of rudimentary uteri may be underestimated. More than 10% of the patients with MRKH syndrome may need surgery to remove the dysplastic uteri during their lifetime. Patients should be emphasized on the need for long-term follow-up and vigilance for obstructive symptoms.

For evaluation and management of the rudimentary uteri of MRKH syndrome, assessment of “functionality” by focusing on clinical blocked bleeding symptoms is highly recommended due to limitations of imaging and/or pathological examination. For patients with obstructive abdominal pain, laparoscopic exploration and prophylactic hysterectomy are recommended, regardless of the positive or negative results of image examination. The underlying mechanism of delayed symptomatic attack needs further studies, including development and improvement of imageological techniques.

## Data Availability

The clinical data used to support the findings of this study are included within the article.

## Abbreviations

MRKH: Mayer–Rokitansky–Küster–Hauser (syndrome)
PUMCH: Peking Union Medical College Hospital
MRI: Magnetic resonance imaging
VAS: Visual analog scale
CIs: Confidence intervals
CDFI: Color Doppler flow imaging.
